# Characterization of *Aspergillus terreus* Accessory Conidia and Their Interactions With Murine Macrophages

**DOI:** 10.3389/fmicb.2022.896145

**Published:** 2022-06-16

**Authors:** Isabell Henß, Christoph Kleinemeier, Lea Strobel, Matthias Brock, Jürgen Löffler, Frank Ebel

**Affiliations:** ^1^Institute for Infectious Diseases and Zoonoses, Ludwig-Maximilians-University Munich, Munich, Germany; ^2^Department of Internal Medicine II, University Hospital of Wuerzburg, Wuerzburg, Germany; ^3^Fungal Genetics and Biology, School of Life Sciences, University of Nottingham, Nottingham, United Kingdom

**Keywords:** galactomannan, accessory conidia, aleurioconidia, dectin-1, β-glucan, desiccation, *Aspergillus terreus*

## Abstract

All *Aspergillus* species form phialidic conidia (PC) when the mycelium is in contact with the air. These small, asexual spores are ideally suited for an airborne dissemination in the environment. *Aspergillus terreus* and a few closely related species from section *Terrei* can additionally generate accessory conidia (AC) that directly emerge from the hyphal surface. In this study, we have identified galactomannan as a major surface antigen on AC that is largely absent from the surface of PC. Galactomannan is homogeneously distributed over the entire surface of AC and even detectable on nascent AC present on the hyphal surface. In contrast, β-glucans are only accessible in distinct structures that occur after separation of the conidia from the hyphal surface. During germination, AC show a very limited isotropic growth that has no detectable impact on the distribution of galactomannan. The AC of the strain used in this study germinate much faster than the corresponding PC, and they are more sensitive to desiccation than PC. During infection of murine J774 macrophages, AC are readily engulfed and trigger a strong tumor necrosis factor-alpha (TNFα) response. Both processes are not hampered by the presence of laminarin, which indicates that β-glucans only play a minor role in these interactions. In the phagosome, we observed that galactomannan, but not β-glucan, is released from the conidial surface and translocates to the host cell cytoplasm. AC persist in phagolysosomes, and many of them initiate germination within 24 h. In conclusion, we have identified galactomannan as a novel and major antigen on AC that clearly distinguishes them from PC. The role of this fungal-specific carbohydrate in the interactions with the immune system remains an open issue that needs to be addressed in future research.

## Introduction

We currently know of ~300 *Aspergillus* species (Houbraken et al., 2014). *Aspergillus terreus* is a prominent example of the few species that are pathogenic to humans or animals, and this species is also a relevant plant pathogen (Louis et al., 2014). Two characteristic features of this mold are of particular importance for its virulence, namely, (1) its intrinsic resistance to amphotericin B and (ii) its highly developed ability to cause disseminating infection (Walsh et al., 2003; Steinbach et al., 2004). Infections commonly start with the inhalation of phialidic conidia (PC). A distinctive feature of members of the *A. terreus* species complex is their ability to form a second type of asexual spores, the accessory or aleurioconidia (AC) (Lass-Flörl et al., 2021). In comparison to PC, AC are larger and have a lower membrane ergosterol content. Whether the latter is the reason for the enhanced resistance of *A. terreus* to the ergosterol-binding drug amphotericin B is still a matter of discussion (Lass-Flörl et al., 2005; Deak et al., 2009).

AC and PC are formed under different conditions and most likely serve different functions. PC arise from conidiophores, specialized structures that are formed if the fungus is in direct contact to the air. The principal function of PC is to allow an efficient, airborne distribution in the environment. Consequently, these spores must be able to endure prolonged times under conditions that are characterized by UV light, desiccation, and oxidative stress. The layers of melanin and hydrophobins are structural elements of PC that are important in this context. AC lack these features and emerge from hyphae during growth in a liquid environment (Deak et al., 2009). They are also produced during infection and have been detected in infected murine tissue, *Galleria mellonella* larvae, and potato leaves (Seligsohn et al., 1977; Slesiona et al., 2012a,b; Louis et al., 2014; Lackner et al., 2019). This led to the hypothesis that AC formation during infection may facilitate the dissemination of the fungus and result in increased mortality rates. To verify this, Lackner et al. (2019) analyzed 15 *A. terreus* strains and found no evidence for a higher virulence of AC compared to PC in a *G. mellonella* model of infection. Deak et al. (2011) showed that AC trigger a much stronger inflammatory response in infected macrophages and a murine model of pulmonary aspergillosis compared to PC. The same authors detected distinct patches of β-glucan on the surface of AC and reasoned that the exposure of this major fungal pathogen-associated molecular pattern (PAMP) may be responsible for the strong immune response (Deak et al., 2011).

In this study, we have characterized AC of a novel clinical isolate of *A. terreus* and analyzed the interactions of these specialized spores with murine J774 macrophages. Our data demonstrate that AC differ from PC in many aspects and indicate that AC have the potential to play an important role during infection.

## Results

We have isolated a filamentous fungus from the kidney of a dog that had died from a systemic mycosis. The colonies of this strain showed a light brown color and *Aspergillus*-like conidiophores (data not shown). Sequencing of the ITS1/ITS2 locus and the β-tubulin gene identified it as *A. terreus* (data not shown). The strain was designated At17-14 and deposited in the DSMZ strain collection (DSM 113823).

**Figure 1:**
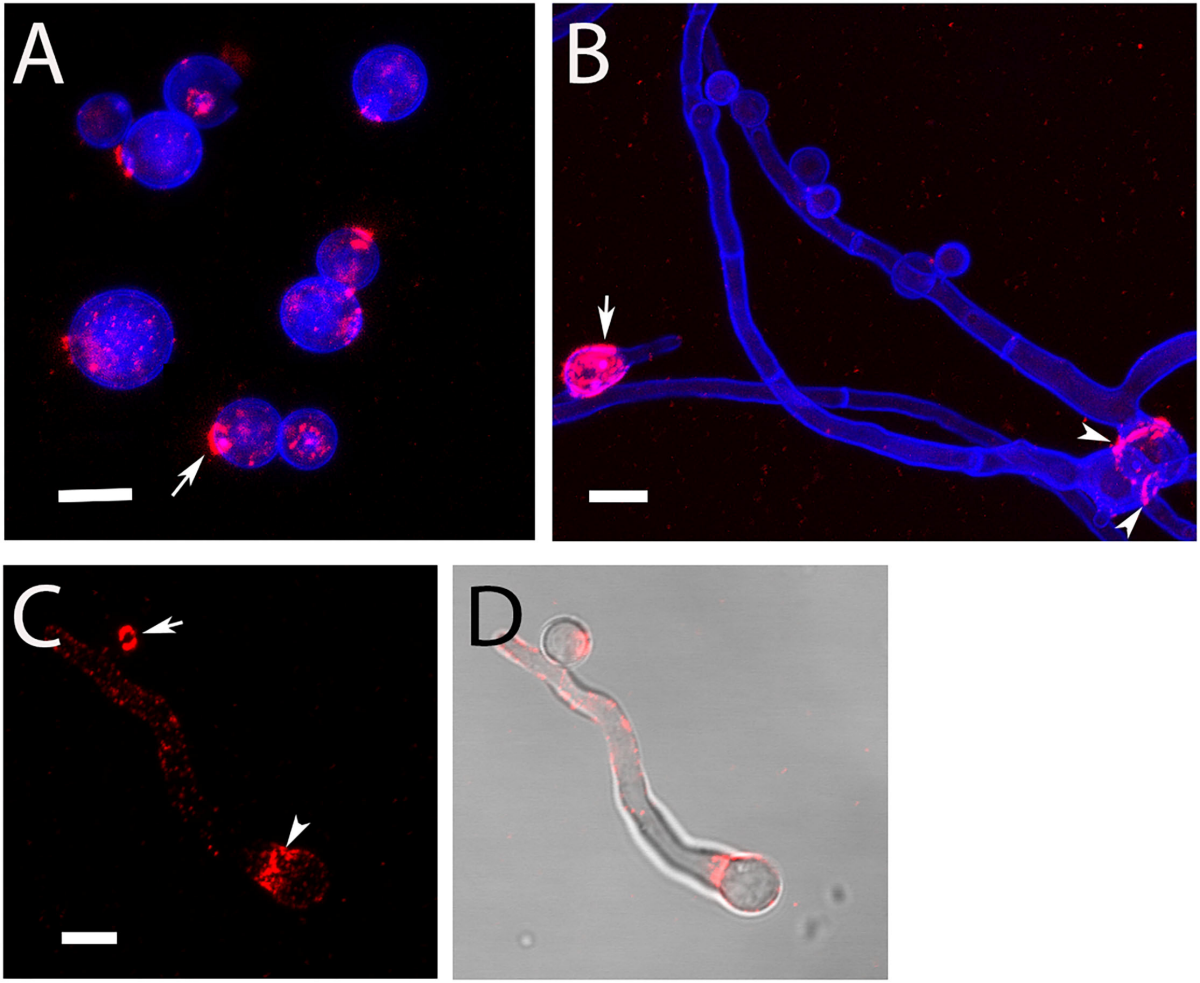
Detection of surface-accessible β-glucan on AC and hyphae of strain At17-14. β-Glucan was stained using mab 2G8 (red). The fungal cell wall was additionally visualized with the chitin-specific dye Calcofluor white (blue). Arrows in **(A,C)** indicate ring-like, β-glucan-positive structures. The arrow in **(B)** points a conidial body with a patchy staining of the entire surface. The arrowheads in **(B,C)** indicate a collar-like pattern of β-glucan in the proximal part of the emerging germ tube. **(D)** Shows an overlay of the galactomannan staining shown in **(C)** and a bright field image. Bars in **(A–C)** represent 5μm.

During submerse culture, AC emerged from the hyphae of At17-14. The production of AC agrees with the strain identification as it represents another characteristic feature of *A. terreus* (Deak et al., 2009; Lass-Flörl et al., 2021). We isolated AC and analyzed them by immunofluorescence microscopy. Monoclonal antibody (mab) 2G8 that is specific for β-glucan (Torosantucci et al., 2005) stained distinct dots and prominent circular structures on the conidial surface (Figure 1). This pattern is similar to the one reported previously after staining with recombinant dectin-1 molecules (Deak et al., 2011). The ring-like structures share similarities with the scars found on budding yeast. We therefore hypothesized that these structures derive from a breakup of the contact sites of AC with the hyphal body. We indeed observed that AC that were still attached to hyphae were not stained by 2G8 (Figure 1B), which demonstrates that the β-glucans are only exposed after separation from the hypha. After germination, the conidial bodies of AC were stained by 2G8 in two distinct patterns, namely, the β-glucan was either irregularly distributed over the entire conidial surface (Figure 1B, arrow) or the staining was concentrated in collar-like structures at the sites of germ tube emergence (Figures 1B,C, arrowheads).

**Figure 2:**
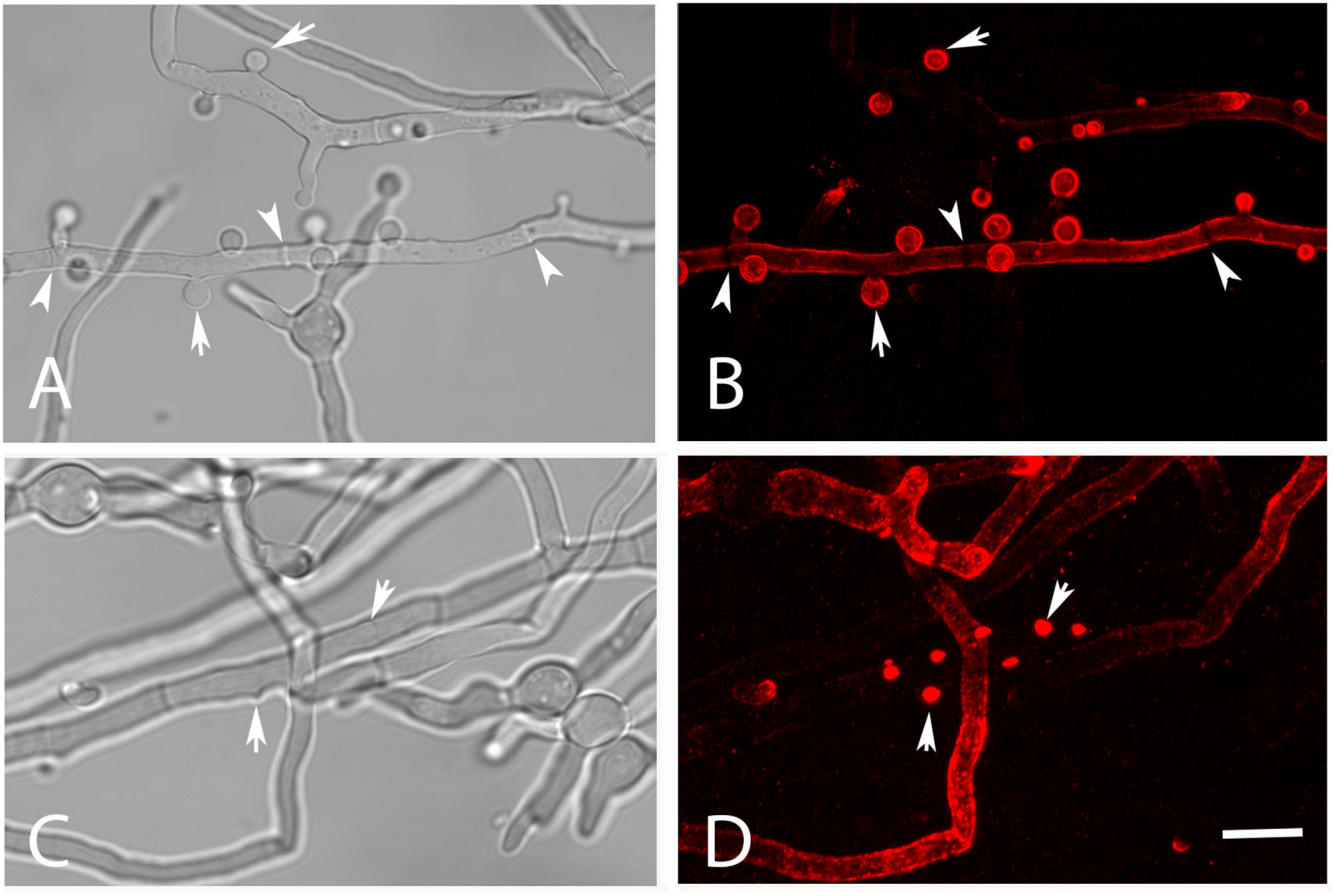
Detection of galactomannan on hyphae of strain At17-14 grown in AMM at 37°C. The staining with the galactomannan-specific antibody L10-1 is shown in red. Mature AC are indicated by arrows in **(A,B)**. Nascent AC are indicated by arrows in **(C,D)**. Arrowheads point to the positions of septa in **(A,B)**. The bar in **(D)** represents 10μm and is valid for all panels.

Galactomannan is another important component of the *Aspergillus* cell wall and also supposed to function as a fungal PAMP (Garlanda et al., 2002; Serrano-Gómez et al., 2004; Chiodo et al., 2014; Steger et al., 2019). We have analyzed AC for the presence of this carbohydrate using mab L10-1 (Heesemann et al., 2011). This antibody stained the surface of AC in a strong and homogenous fashion (Figures 2A,B); 92.9% (±2.6%) of isolated AC of strain At17-12 were strongly galactomannan-positive. Nascent AC on the hyphae were L10-1-positive at a very early stage when they were hardly visible by light microscopy (Figures 2C,D, arrows). In comparison to AC, the hyphae of At17-14 showed a more variable and often weaker galactomannan staining (Figures 2B,D). Of note, we observed a small galactomannan-negative strip on the hyphal surface at the positions of septa (Figure 2B, arrowheads), a pattern that we have previously also found for *Aspergillus fumigatus* (data not shown). This demonstrates that the cell wall at the site of the septum differs from the remaining hyphal cell wall.

To confirm that the presence of large amounts of galactomannan is a common feature of *A. terreus* AC, we analyzed hyphae of the *A. terreus-*type strains T9 and SBUG844. Both strains produced AC that were strongly labeled by L10-1 (Supplementary Figure 1).

**Figure 3:**
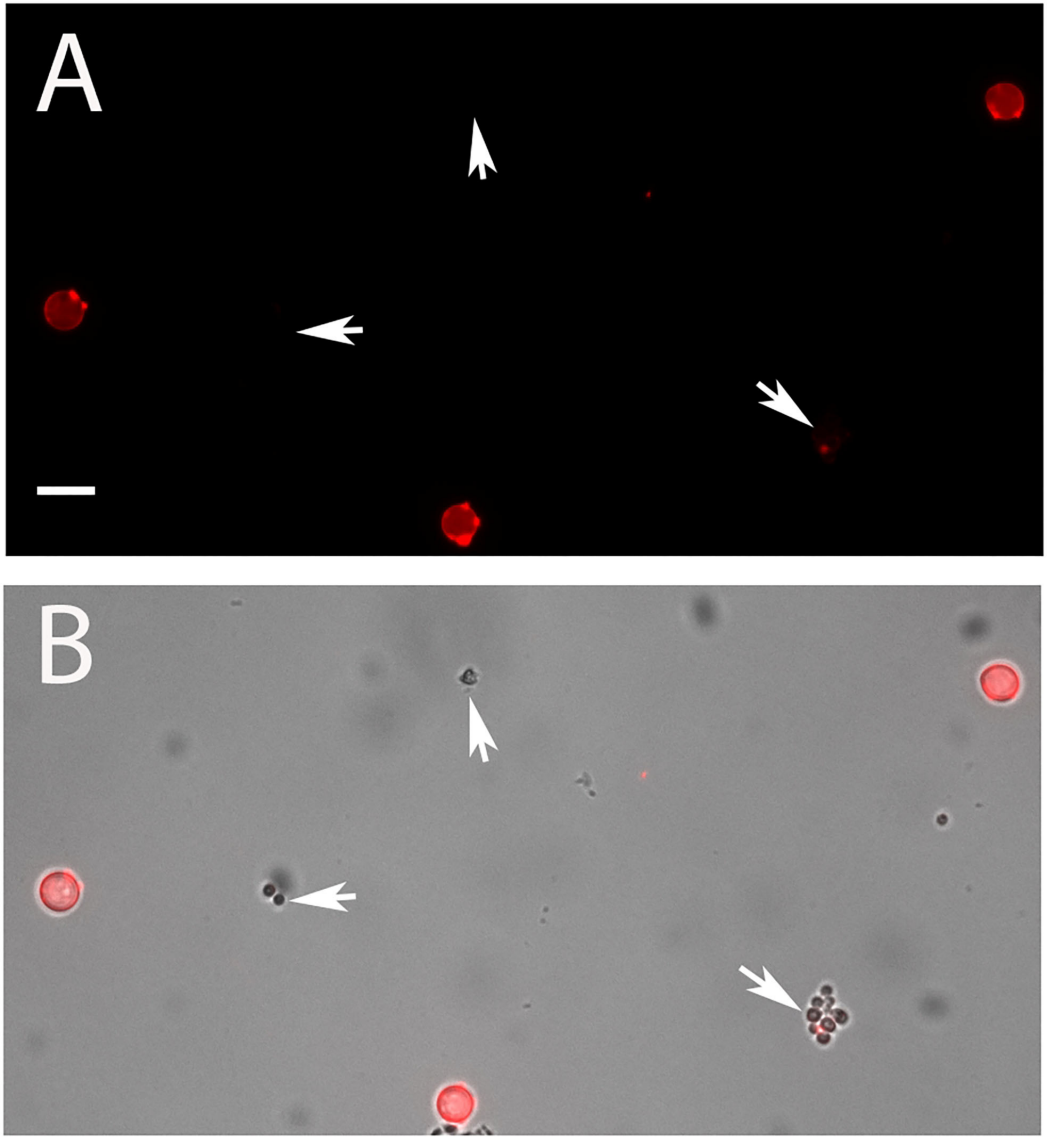
AC and PC of strain At17-14 were stained in red with the galactomannan-specific antibody L10-1. **(B)** shows the overlay of a galactomannan staining and the corresponding bright field image. PC are indicated by arrows. The bar in **(A)** represents 5μm and is valid for all panels.

In the next step, we compared isolated PC and AC of strain At17-14 and the two reference strains NIH2624 and SBUG844. All AC were strongly galactomannan-positive, whereas PC showed only a very weak staining that was hardly detectable using the settings that resulted in a strong staining of AC. This pattern was found with all strains. A representative image of strain At17-14 is shown in Figure 3, which also demonstrates that AC are clearly larger than PC.

**Figure 4:**
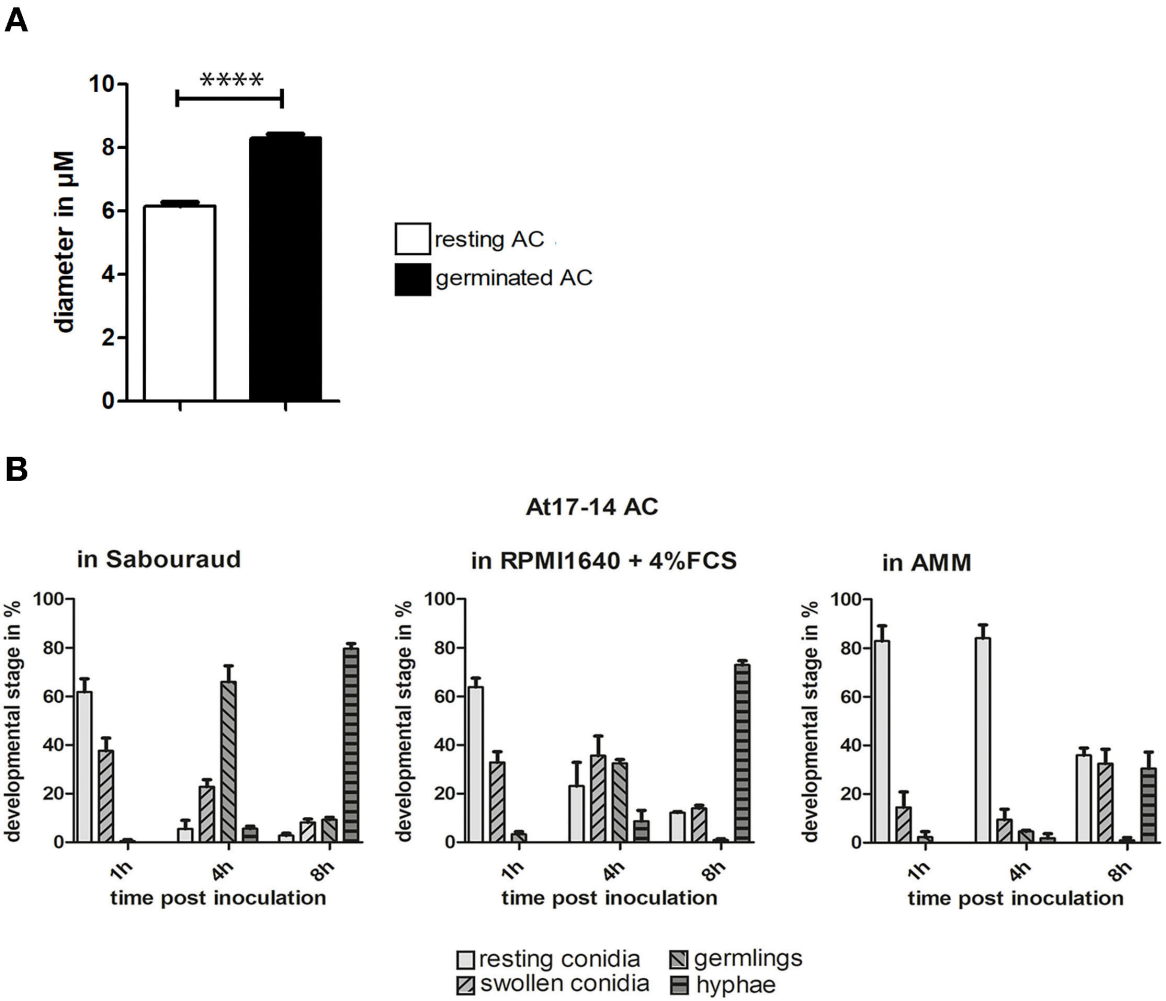
Germination of AC. **(A)**: the diameters of AC of strain At17-14 were measured for resting conidia and conidial bodies after germination in cell culture medium. Each column represents the values of three groups comprising 50 measurements each; standard deviations are indicated. ****p < 0.0001 according to an unpaired Student's *t*-test. **(B)** shows a quantitative analysis of the germination process over time for AC of strain At17-14 in three different media (AMM, Sabouraud, and RPMI1640 + 5% FCS). Each column represents the values of three groups each comprising 60 events. Standard deviations are indicated.

The swelling of conidia is a hallmark of the early germination process of *Aspergillus* PC. To analyze this parameter for AC, we compared the diameter of resting AC and AC after germ tube formation. We found that the average diameter increased by approximately one-third (Figure 4A), indicating that the isotropic growth of AC is less pronounced compared to that of *Aspergillus* PC that increase by a factor of 1.5–3 (Rohde et al., 2002). We also analyzed the speed of the germination process in three different media, namely, Aspergillus Minimal Medium (AMM), Sabouraud, and RPMI1640 cell culture medium (the latter contained 5% fetal calf serum). Germination of AC was fastest in Sabouraud medium and slowest in AMM. After 8 h in Sabouraud nearly 80% of AC had formed hyphae compared to only 20% in AMM (Figure 4B).

A comparison of the germination of AC and PC of strain At17-14 and PC of *A. fumigatus* strain D141 and *A. terreus* strain SBUG844 revealed the fastest germination for At17-14 AC. PC of *A. fumigatus* strain D141 were only slightly slower, whereas the germination of PC of the two *A. terreus* strains was much slower (Supplementary Figure 2).

**Figure 5:**
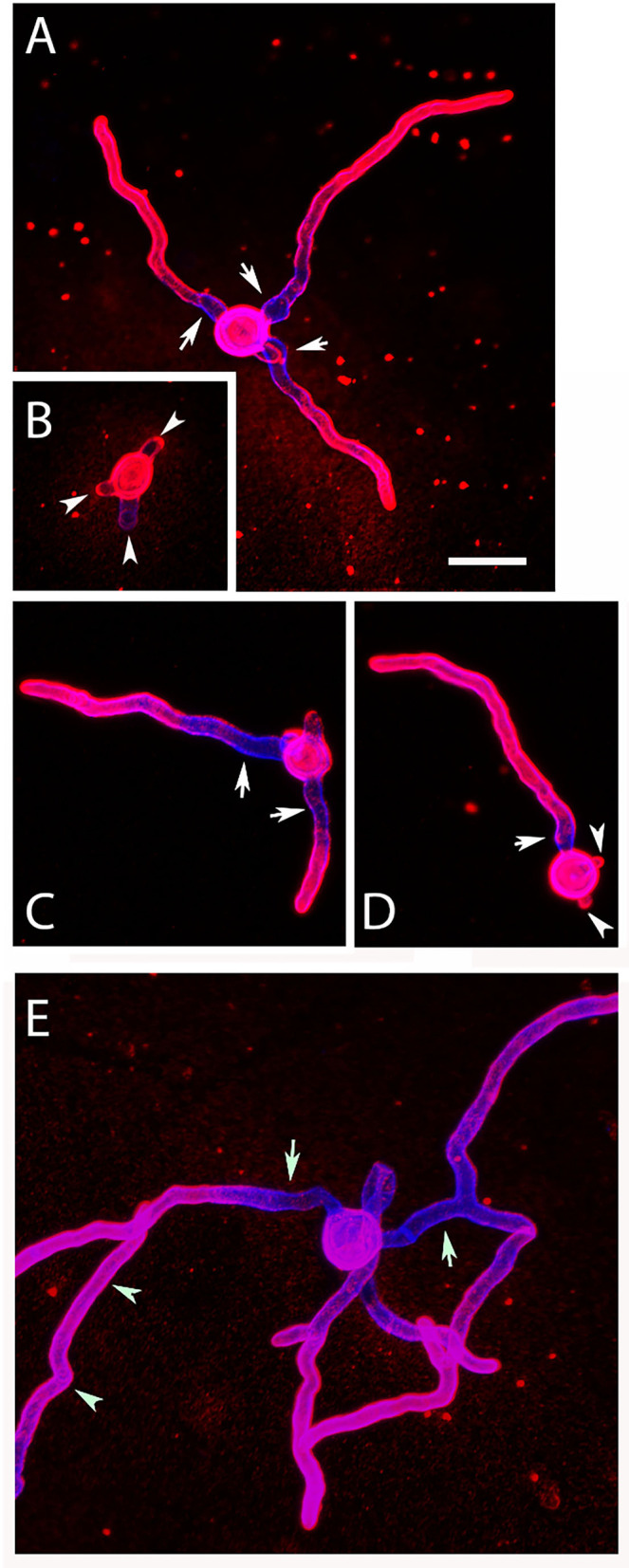
Germination of AC: morphology and distribution of galactomannan. Purified AC were cultivated at 37°C in AMM, fixed after 6 and 9 h, and stained with Calcofluor white (blue) and the galactomannan-specific antibody L10-1 (red). Arrows in **(A,C–E)** indicate hyphal regions proximal to the conidial bodies showing a weaker galactomannan staining. The arrowheads in **(B,D)** indicate sites of emerging germ tubes. The arrowheads in **(E)** mark a hypha that is not part of the small hyphal network originating for the central AC. The bar in **(A)** represents 10μm and is valid for all panels.

To analyze changes in morphology and the distribution of galactomannan during germination, AC were incubated in RPMI1640 cell culture medium at 37°C. After 6 h, many AC formed short hyphae and initiated a simultaneous formation of multiple germ tubes (Figures 5B,D, arrowheads). This is particularly evident in a stack of confocal images of an individual AC shown in Supplementary Figure 3. Apart from the initial germ tube (indicated by an arrow in D), the set of images show three additional germination sites in an early stage (Supplementary Figures 3A,F, arrowheads), indicating that AC can initiate a simultaneous formation of multiple germ tubes in a synchronized manner. This enabled them to establish branched networks of short hyphae after 9 h (Figure 5E). Most parts of the hyphae were strongly stained with L10-1, but a short stretch proximal to the conidial bodies showed a much weaker galactomannan staining (Figures 5A,C, arrows).

**Figure 6:**
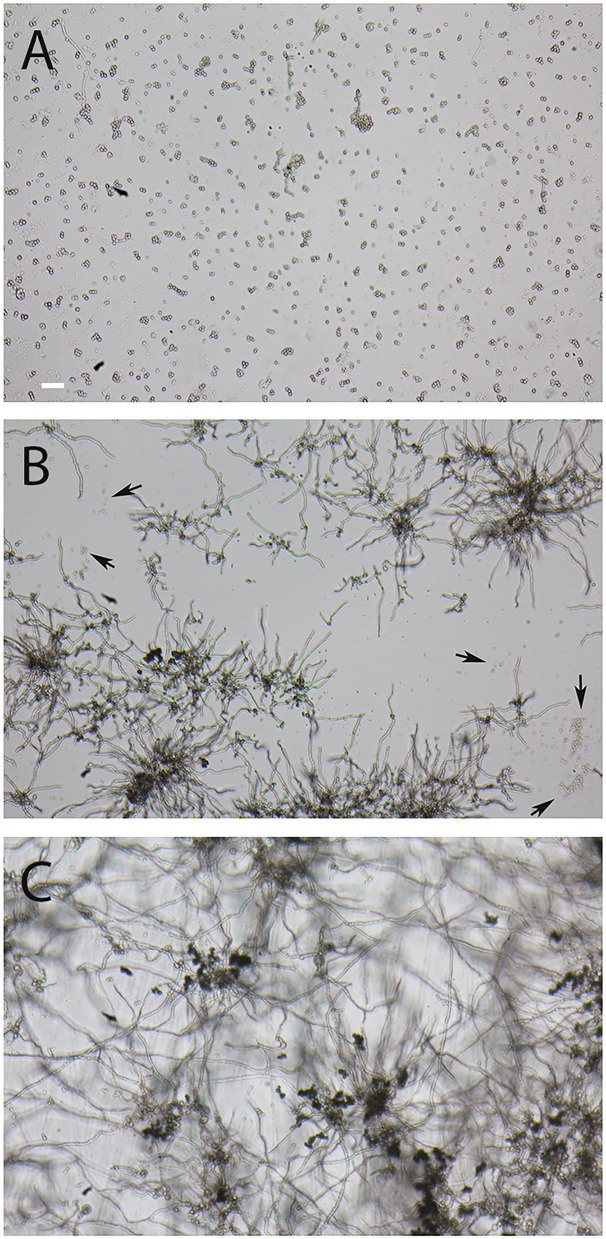
Comparison of the resistance of AC and PC to desiccation. Small droplets of conidial suspension were placed on the surface of Petri dishes and desiccated for 16 h at 37°C. The conidia were then covered with Sabouraud medium and further incubated for 14 h at 37°C. **(A)** shows AC of strain At17-14, the corresponding PC are depicted in **(B)**. Arrows indicate clusters of At17-14 PC showing no signs of activation. **(C)** shows PC of the *A. fumigatus* strain D141for comparison. The bar in **(A)** represents 50μm and is valid for all panels.

AC are produced by *A. terreus* hyphae during submerse culture, whereas PC are formed on conidiophores that are directly exposed to the air. PC are distributed through the air, while AC are more likely to be released into an aqueous environment. Considering this, it was likely that AC and PC differ in their ability to tolerate desiccation. To test this, we suspended freshly isolated spores in water, positioned small droplets on the surface of Petri dishes, and incubated them at 37°C. Microscopic inspection revealed that all droplets had evaporated after 1 h. After 16 h at 37°C, the desiccated conidia were resuspended in Sabouraud medium and incubated at 37°C for 14 h. A majority of the PC of *A. terreus* and *A. fumigatus* were able to germinate after desiccation (Figures 6B,C), although some *A. terreus* PC remained small and inactive (Figure 6B, arrows). In contrast to PC, the vast majority of AC showed no signs of germination (Figure 6A), demonstrating that these spores are particularly sensitive to desiccation. In control experiments, we also checked the viability of AC and PC from the conidial suspensions that were used in the desiccation experiment and found that >98% of the conidia in these three samples had germinated after 14 h in Sabouraud medium.

### Infection of J774 Macrophages With AC and PC of *A. terreus*

**Figure 7:**
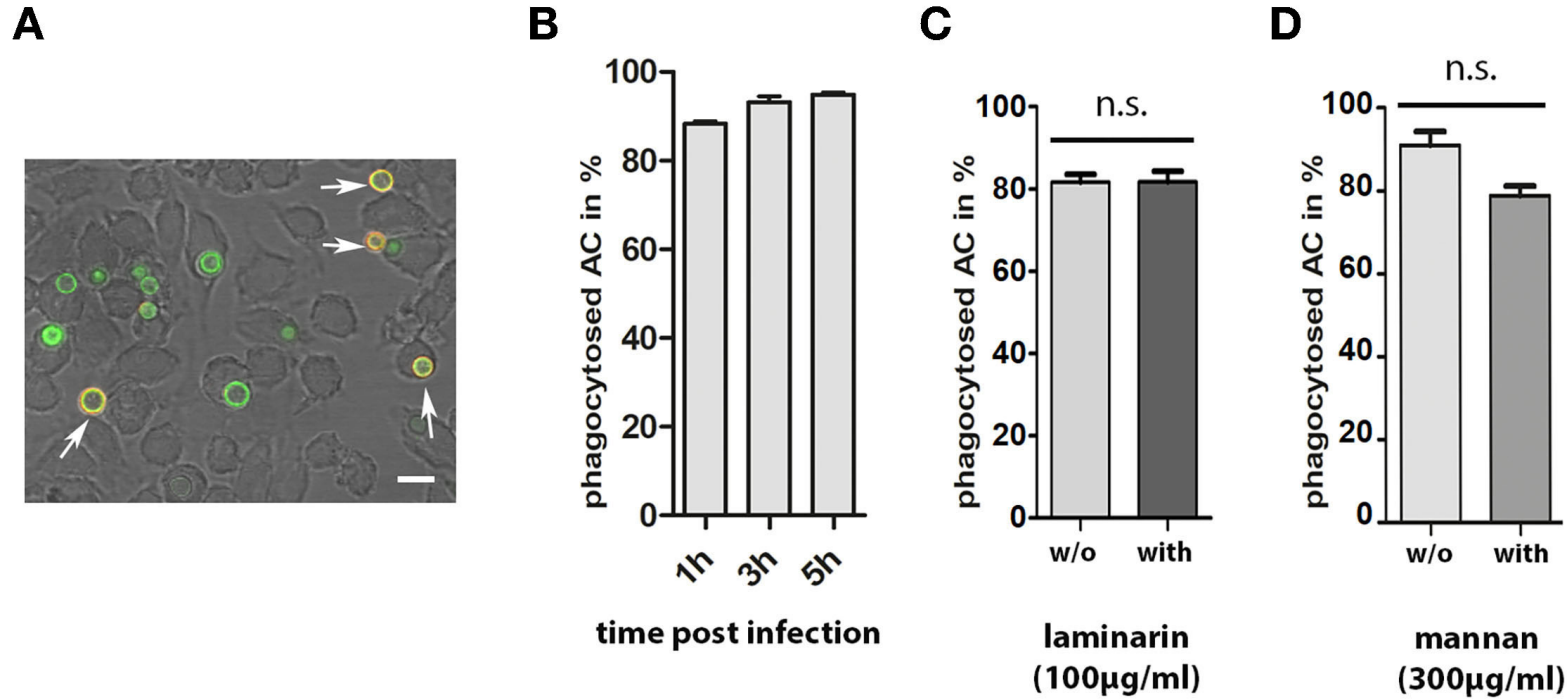
Phagocytosis of AC by J774 macrophages. J774 cells were infected with one FITC-labeled AC per two J774 cells. After 1, 3, and 5 h of co-incubation, samples were fixed and extracellular AC were stained with mab L10-1. A representative image is shown in **(A)**. Extracellular conidia are indicated by arrows. The bar represents 10μm. **(B)** shows the percentage of internalized AC at three different time points. Each column represents the values of four groups with 100 events each. Parallel experiments were performed in the presence of laminarin (100μg/ml) and mannan (300μg/ml) (**C**,**D**, respectively). In these experiments, cells were fixed after 1 h. Each column represents the values of three groups with 100 events each.

During infection, conidia are taken up by phagocytes, such as macrophages. To analyze the interactions between AC and macrophages, we labeled AC with FITC and used them to infect J774macrophages. Samples were fixed 1, 2, and 3 h post infection and extracellular AC were stained with mab L10-1 (Figure 7A). After 1 h, 88% of AC were internalized by the macrophages; this percentage increased slightly to 95% after 5 h (Figure 7B). Hence, phagocytosis of AC by J774 cells is fast and efficient.

The presence of β-glucan and galactomannan on the surface of AC are factors that may be relevant for an efficient phagocytosis. To analyze this, we compared phagocytosis in the presence and absence of laminarin and mannan. Soluble β-glucan from the brown algae *Laminaria digitate* was previously shown to block the dectin-1-mediated phagocytosis of *A. fumigatus* conidia (Luther et al., 2007) and mannan was shown to inhibit the phagocytosis of yeast cells (Taylor et al., 2004). Laminarin (100μg/ml) had no impact on phagocytosis (Figure 7C). The presence of mannan (300μg/ml) led to a minor, but not significant, reduction in the percentage of internalized conidia (Figure 7D).

**Figure 8:**
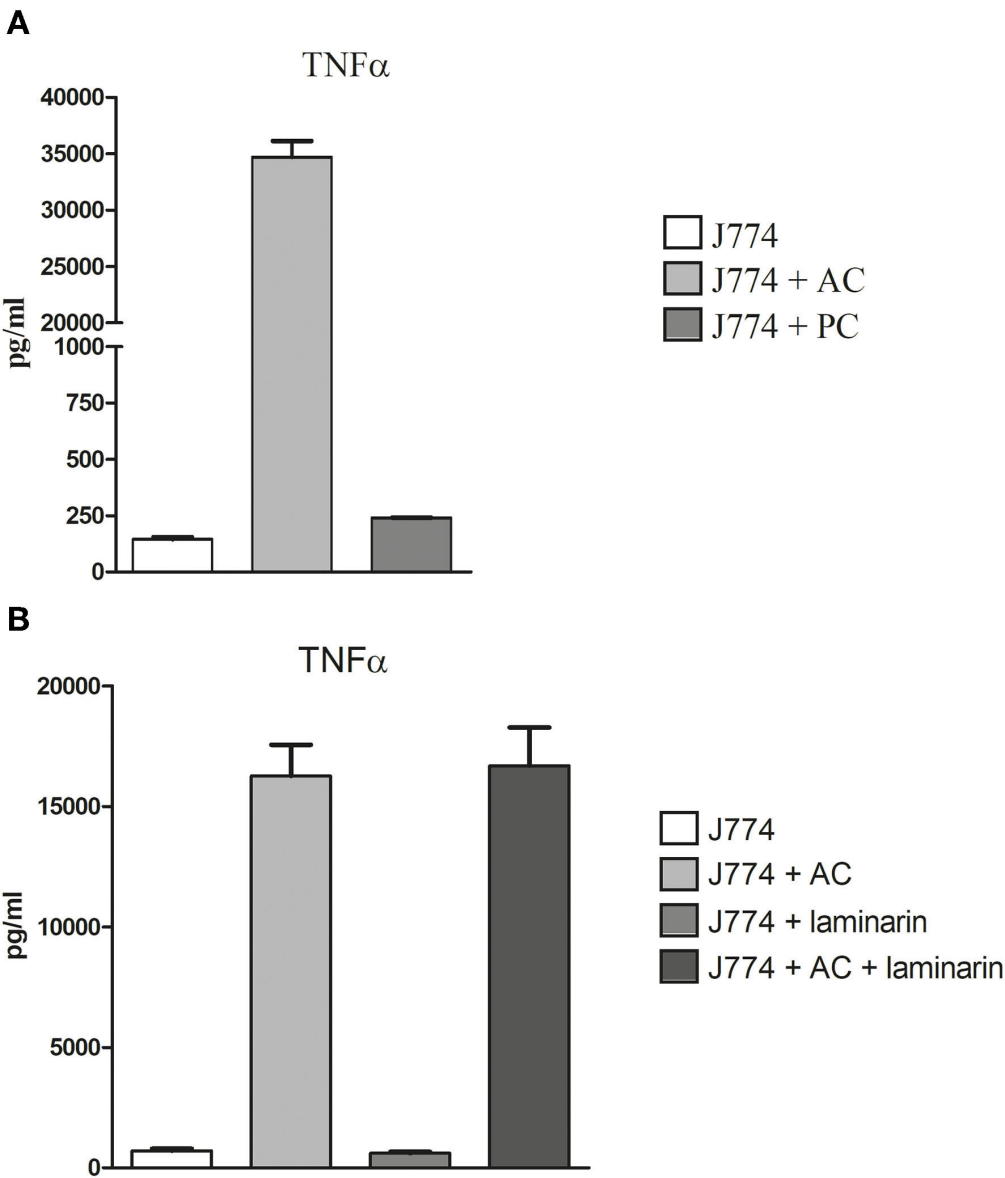
Production of TNFα in AC- and PC-infected J774 macrophages. Macrophages were challenged with 2 AC and 10 PC per macrophage **(A)**. Blocking experiments were performed in the presence or absence of 100μg/ml of laminarin **(B)**. After 6 h, supernatants were harvested and the TNFα concentration was determined by ELISA. Each condition was analyzed in triplicate. Standard deviations are indicated.

Deak et al. (2011) showed that alveolar macrophages respond to the presence of AC with a strong production of cytokines. We compared the production of tumor necrosis factor-alpha (TNFα) by macrophages infected for 6 h with either PC or AC of strain At17-14. We observed a strong increase in the production of TNFα for AC- but not for PC-infected macrophages (Figure 8A). Deak et al. (2011) speculated that the inflammatory response was triggered by the β-glucan present on the surface of AC. To test this hypothesis, we infected J774 cells in the presence of laminarin. Laminarin had no impact on the TNFα release of infected or non-infected J774 cells, indicating that the inflammatory response to AC is not essentially triggered by the β-glucan present on the conidial surface (Figure 8B), which agrees with the undisturbed phagocytosis in the presence of laminarin (Figure 7C).

**Figure 9:**
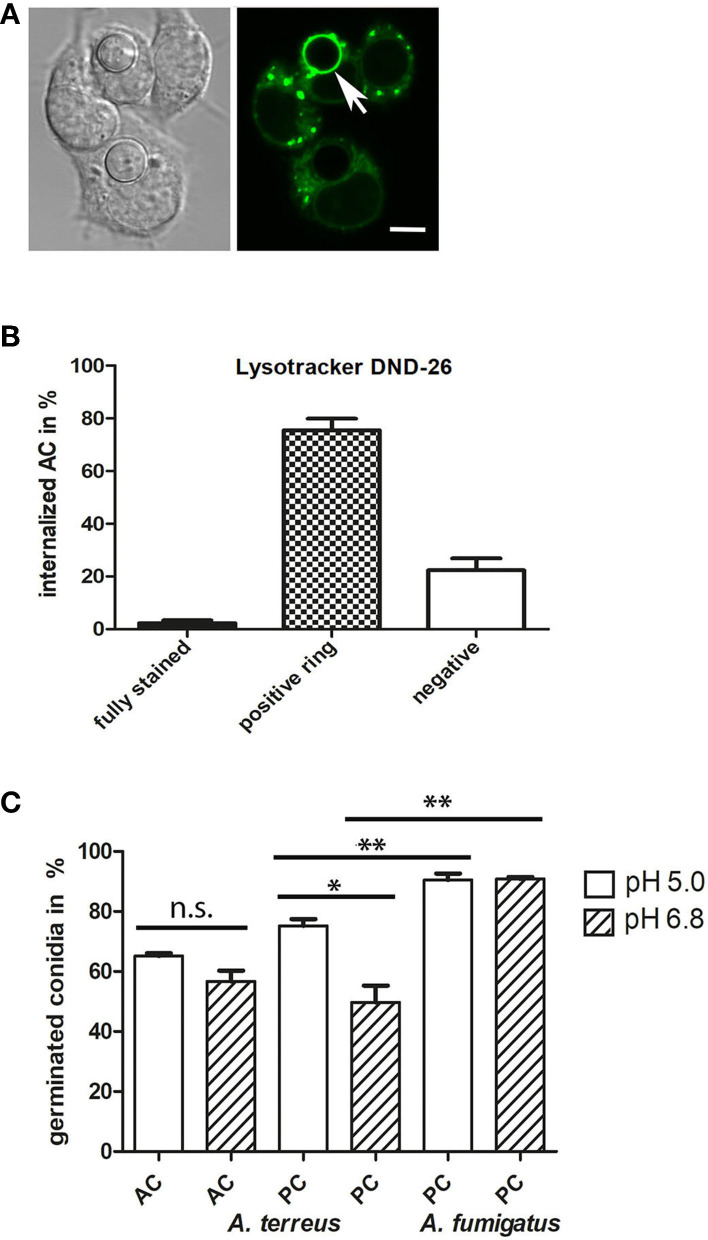
Internalized AC reside in acidified vacuoles. J774 cells were infected with one AC per two J774 cells. After 6 h of infection, LysoTracker DND-26-stained samples were analyzed by live-cell microscopy. A representative image is shown in **(A)**; an AC within an acidified vacuole is indicated by an arrow. The bar represents 5μm. **(B)** shows a quantitative evaluation of AC that were either directly stained by the LysoTracker (fully stained), resided in acidified vacuoles (positive ring), or were not associated with an acidified compartment (negative). **(C)** shows a quantification of germinated AC (from strain At17-14) and PC (from strains At17-14 and *A. fumigatus* D141) in AMM adjusted to a pH of 5.0 or 6.8. Germination was determined after 7 h for AC and 14 h for PC. Each condition was analyzed in triplicate. Standard deviations are indicated. **p < 0.01, * *p* < 0.1. n.s., not significant.

To investigate the intracellular fate of AC, we challenged J774 cells with AC and stained acidified phagolysosomes using LysoTracker DND-26 (a representative image is shown in Figure 9A). Stacks of confocal images were generated 6 h post infection. Approximately 75% of the macrophage-associated AC were found in LysoTracker-positive vacuoles that were visible as green rings around the AC, 20% of the AC showed no association with LysoTracker-positive vacuoles (Figure 9B) and 2% of the AC were itself completely labeled by the dye, indicating an acidification of the cytoplasm of these spores, most likely as a result of an antimicrobial effector mechanism.

The pH in the phagolysosomes of J774 macrophages was previously determined to be approximately 5 (Kuehnel et al., 2001). To study the impact of the pH on germination, we adjusted AMM to pH 6.8 and pH 5.0, inoculated the samples with conidia of *A. terreus* At17-14 (AC and PC) and *A. fumigatus* D141 (PC), and incubated them at 37°C. After 7 h for AC and 14 h for PC, microscopic images were taken and the percentage of germinated conidia was determined. As shown in Figure 9C, germination of AC and *A. fumigatus* PC was not significantly affected by the pH of the medium. Both types of conidia showed a roughly comparable germination rate, but it needs to be mentioned that germination was analyzed at different time points, after 7 h for AC and 14 h for PC. As expected, PC of *A. fumigatus* showed an increased germination rate compared to *A. terreus* PC (Slesiona et al., 2012a). Germination of *A. terreus* PC was faster under the more acidic conditions, but did not reach the level observed for *A. fumigatus* PC (Figure 9C).

**Figure 10:**
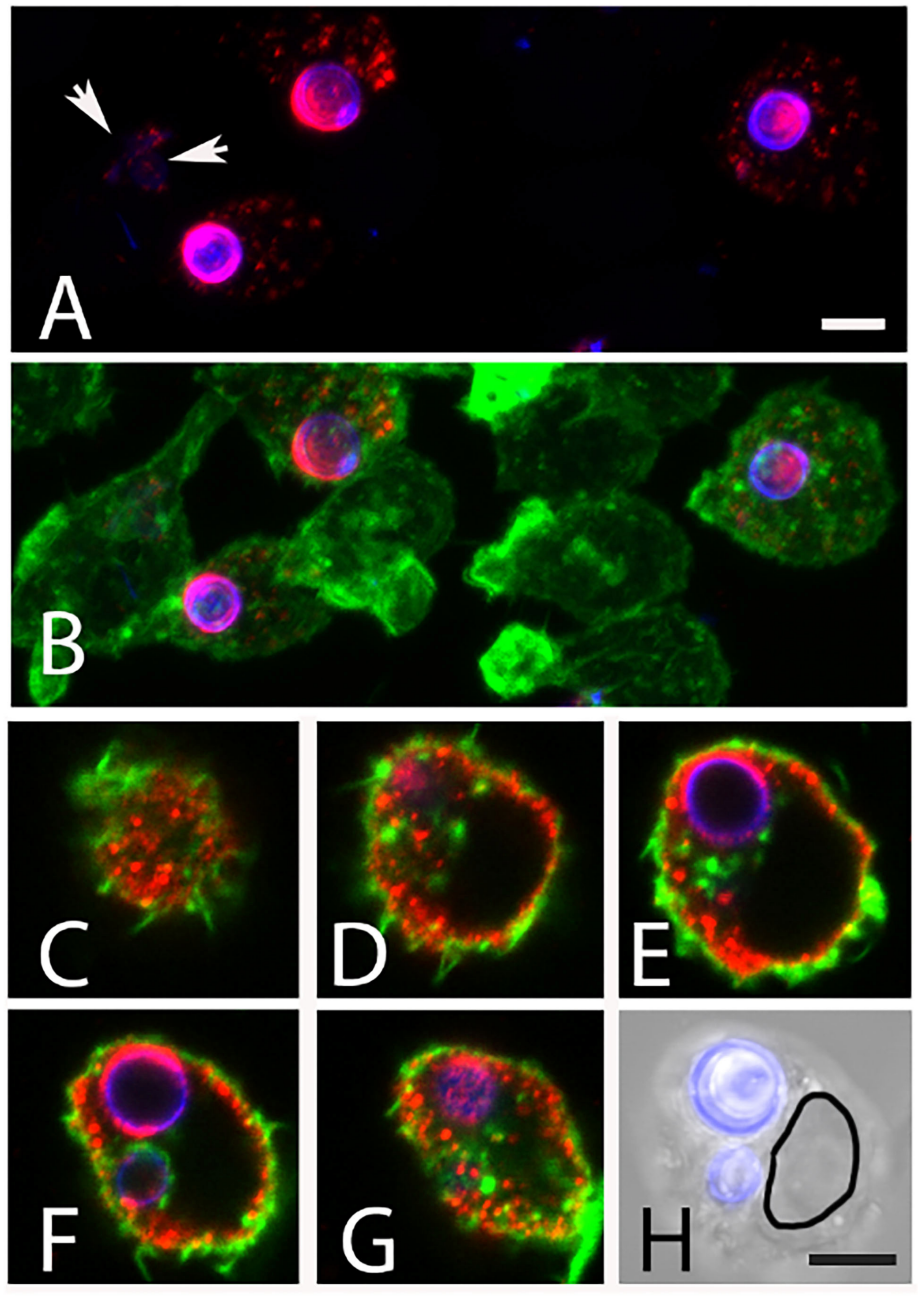
Release of galactomannan from internalized AC. The J774 macrophage cells are visualized using phalloidin-FITC (green). The AC were stained with Calcofluor white (blue) and mab L10-1 (red). **(A,B)** show maximum intensity projections of J774 macrophages after 5 h of co-incubation with AC. Released galactomannan is detectable in the infected cells. The arrow indicates galactomannan in a neighboring, non-infected cell. **(C–G)** represent single optical planes of an infected cell after 6 h. The planes have a distance in Z of 2.8μm. **(H)** shows an overlay of a brightfield image and the Calcofluor white staining of the same cell. The outline of the nucleus is indicated in **(H)**. The bars in **(A,H)** represent 5μm and are valid for [**(A–H)**, respectively].

**Figure 11:**
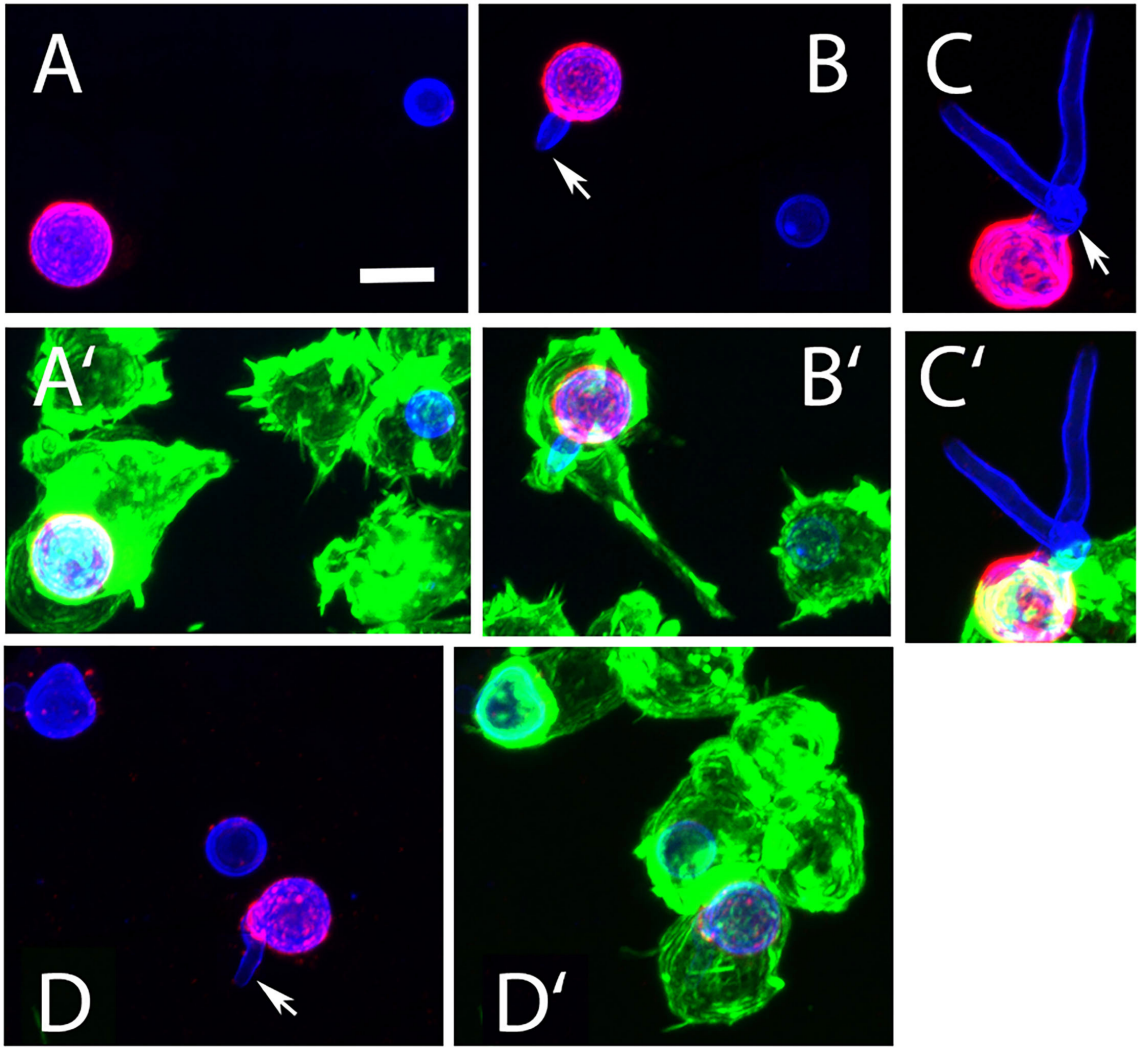
Detection of β-glucan in J774 cells infected with AC. The macrophage cells were infected for 6 h, permeabilized with Triton X100, and visualized using phalloidin-FITC (green) AC were stained with Calcofluor white (blue) and the β-glucan-specific mab 2G8 (red). **(A′–D′)** show overlays of all three colors, the corresponding images without the green channel are depicted in **(A–D)**. All panels show maximum intensity projections. The arrows in **(B–D)** indicate AC that had initiated germ tube formation and were strongly stained with 2G8. The bar in **(A)** represents 5μm and is valid for all panels.

After 6 h of infection of J774 cells, most intracellular AC remained round (Figures 10A,B), whereas extracellular AC present in the same well had established small hyphal networks (data not shown). The intracellular AC retained galactomannan on their surface (Figures 10A,B), but we often observed also small, galactomannan-positive structures in the cytosol and in some rare cases even in neighboring cells (Figure 10A, arrows). A stack of confocal images of an infected macrophage is shown in Figures 10C–G and demonstrates that the galactomannan-positive structures resided in the cytoplasm of the host cell and tended to accumulate below the cytoplasmic membrane. It is also evident from these images that the nuclear membranes excluded the galactomannan-positive structures (compare Figures 10E,H). In a control experiment, non-infected J774 cells showed no staining with L10-1 (data not shown). After 6 h of infection, β-glucan was detectable on some, but not all, internalized AC. A particularly strong β-glucan staining was found for those AC that had initiated germ tube formation (Figures 11A–D, arrows). A release of β-glucan from phagocytosed AC was not observed.

**Figure 12:**
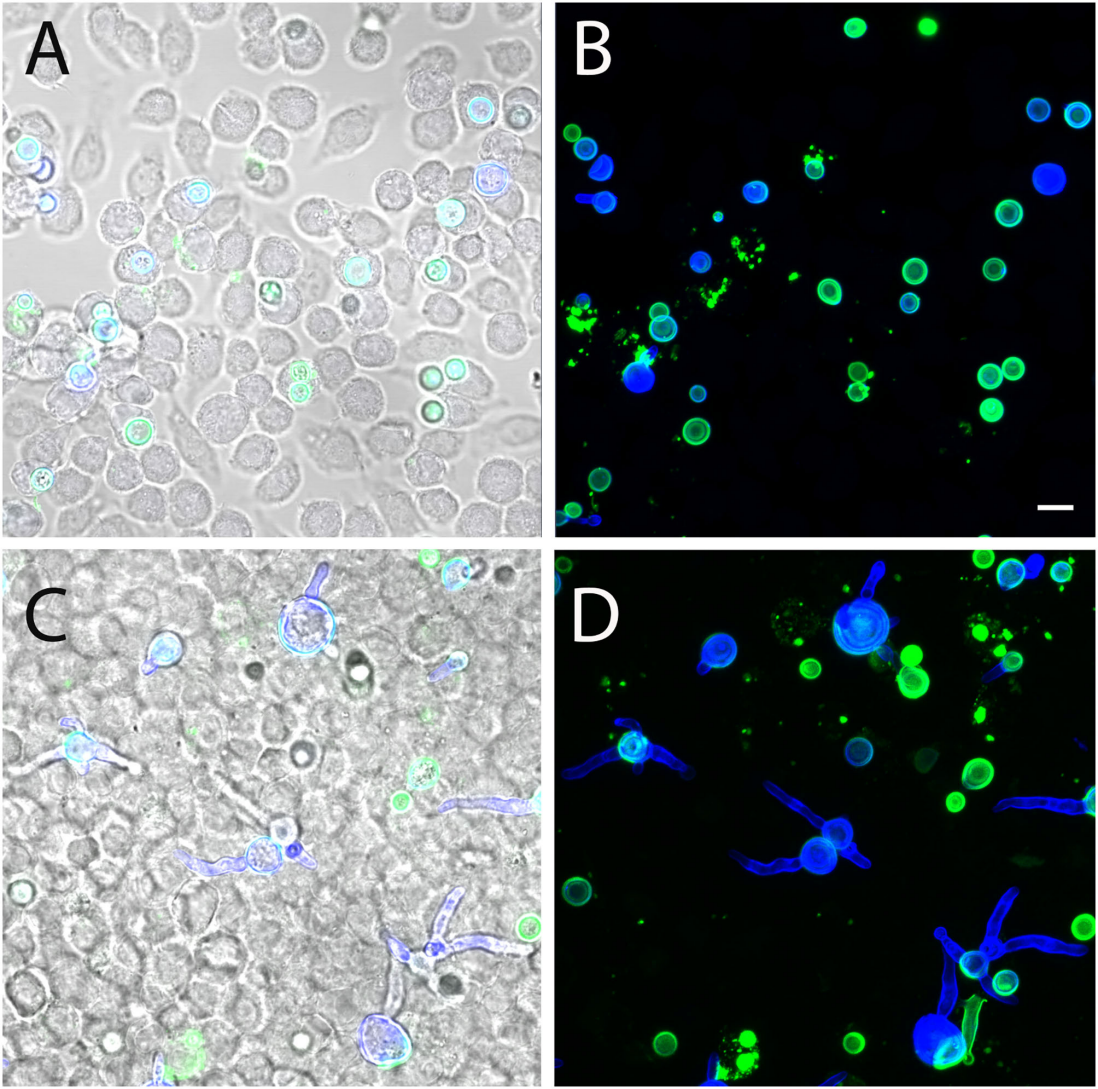
Germination of AC within J774 macrophages. Macrophages were infected with FITC-labeled AC. Samples were fixed after 6 h **(A,B)** and 24 h **(C,D)** and stained with Calcofluor white (blue). **(A,C)** show single optical planes and overlays of the green and blue fluorescence as well as the corresponding brightfield image. **(B,D)** show maximum projects of the green and the blue channel. The bar in **(B)** represents 5μm and is valid for all panels.

To determine whether AC can finally germinate or are killed within infected J774 macrophages, we infected cells with FITC-labeled conidia and replaced the medium after 1 h by fresh medium supplemented with itraconazole to prevent fungal overgrowth. Samples were analyzed 6 and 24 h post infection, and fungal elements were stained using Calcofluor white. After 6 h, most conidia were found in tight association with the macrophages and remained in a resting state (Figures 12A,B). After 24 h, approximately 50% of the AC had initiated germ tube formation (Figures 12C,D). This indicates that AC can survive within J774 macrophages and can overcome the antimicrobial mechanisms employed by these phagocytes.

## Discussion

In the genus *Aspergillus*, the formation of AC is restricted to certain species, particularly *A. terreus* and members of the *A. terreus* species complex (Lackner et al., 2019). In contrast to PC, AC are substantially larger, which argues against a role of these spores as major infectious propagules, at least *via* the oral route. AC lack two characteristics of PC, namely, the melanin and the hydrophobin layer (Deak et al., 2009). This likely renders them more sensitive to stress conditions that are associated with an airborne transmission, e.g., UV light, oxidative stress, and desiccation. In contrast to PC, AC are formed in submersed culture (Deak et al., 2009) and in infected tissue (Seligsohn et al., 1977; Walsh et al., 2003) and may boost fungal dissemination *via* the bloodstream. Based on these properties and their capability to trigger a strong inflammatory response, AC have been discussed as an important virulence trait of *A. terreus* (Deak et al., 2011; Lass-Flörl et al., 2021).

The starting point for this study was the isolation of strain At17-14 from a dog suffering from a systemic mycosis. The isolate was identified as *A. terreus* and showed a particular efficient formation of AC. During *in vitro* culture, AC formation is most prominent at later stages, when nutrients are running short. AC may therefore represent a specialized cell type that allows the fungus to overcome unfavorable conditions.

A strong immune response to AC was previously reported by Deak et al. (2011), which suggests that cell wall-bound PAMPs are recognized by the corresponding pattern recognition receptors. Our data confirm that exposure of macrophages to AC results in a strong TNFα production. This response was unaffected by laminarin, which argues against an important role of β-glucans. The fact that mannan reduced phagocytosis only slightly does not exclude a recognition of galactomannan by more specific receptors that are not blocked by mannan. Alternatively, the inflammatory response could be triggered by additional PAMPs that are most likely also present in the cell wall of AC. The fact that macrophages infected with PC of At17-14 showed no TNFα response after 6 h indicates that these still resting *A. terreus* spores have no PAMPs on their surface and seem to be as immunologically inert as resting *A. fumigatus* PC (Aimanianda et al., 2009). Carbohydrate antigens represent important fungal PAMPs, and β-glucan and galactomannan are prominent examples that were both shown to elicit strong pro-inflammatory immune responses (Vassallo et al., 2000; Chiodo et al., 2014). We used two monoclonal antibodies (mabs) to analyze the distribution of these antigens on AC: 2G8 to detect β-1,3- and β-1,6-glucans (Torosantucci et al., 2009) and L10-1 that recognizes galactomannan (Heesemann et al., 2011). With 2G8, the hyphal surface of At17-14 was either negative or only small and sparsely distributed spots were stained. On ACs, however, 2G8 recognized distinct structures, similar to those previously stained on AC with a soluble form of the β-glucan receptor dectin 1 (Willment et al., 2001; Deak et al., 2011). The β-glucan-positive structures were often ring-like and thereby resembled the budding scars found on yeast cells. AC that were still attached to the hyphal body were not recognized by the β-glucan-specific antibodies, indicating that the dissociation of AC from the hyphal surface results in an exposure of β-glucan antigens. This suggests that these structures were present in the interior of the AC cell wall, but not on its surface. After germination under *in vitro* conditions and within infected macrophages, we observed two redistribution patterns for the β-glucans on AC, namely, the antigens were either spread over the entire surface of the conidial bodies or they were particularly enriched at the sites of germ tube emergence. These redistributions result most likely from cell wall reorganizations. We know that the size, cell wall, and surface of *A. fumigatus* PC are the subject of substantial changes, which accompany the dramatic swelling of the conidial body (Rohde et al., 2002; Dague et al., 2008). In contrast to PC, the diameter of AC increases only slightly during germination. Whether this moderate isotropic growth is able to trigger the observed reorganizations of the β-glucan positive structures remains an open question.

Galactomannan is a major constituent of the *Aspergillus* hyphal cell wall. It is absent from the surface of resting PC and becomes exposed during germination (Heesemann et al., 2011). In this study, we have identified galactomannan as a major antigen on the surface of AC. In contrast to β-glucan, galactomannan is homogenously distributed over the entire conidial surface, whereas it is largely absent from the surface of *A. terreus* PC. Galactomannan is also present on the surface of *A. terreus* hyphae, but the staining was weaker and more irregular compared to AC. Strikingly, we could detect AC at a very early, developmental stage as strongly galactomannan-positive structures, although these nascent spores were hardly visible by light microscopy. Germination of AC had no obvious impact on the presence and distribution of surface-accessible galactomannan. This and the absence of scar-like structures suggest that galactomannan is homogeneously distributed throughout the cell wall of AC.

In microscopic images of germinating AC, we observed that these conidia are able to initiate a simultaneous formation of multiple germ tubes. This may conform to the previously noticed hyperpolarization phenotype of AC (Deak et al., 2011). The fast outgrowth at multiple sites clearly distinguishes AC from PC and enables AC to rapidly form small hyphal networks that, during infection, may prevent phagocytosis. The larger size of AC, compared to PC, likely reflects a higher content of stored nutrients, which may boost the germination process and allow formation of multiple germ tubes.

We also noticed that AC of strain At17-14 showed a much faster germination than the corresponding PC. A comparison with PC of *A. fumigatus* and PC from an additional *A. terreus* strain revealed that the AC germinated slightly faster than the *A. fumigatus* PC and much faster than the PC of *A. terreus*. A clear difference in the speed of germination of *A. terreus* and *A. fumigatus* PC was reported by Slesiona et al. (2012a). Our finding that AC of At17-14 germinate faster than the corresponding PC is in line with an observation of Deak et al. (2011), who investigated AC from two *A. terreus* strains. However, a larger study with AC and PC from 15 strains of the *A. terreus* complex found that germination of AC is often slower than that of the corresponding PC (Lackner et al., 2019). Hence, cultivation conditions and strain-specific differences may influence the speed of the germination process and it will be interesting to identify these strain-specific properties.

In the second part of this study, we analyzed the interactions of AC with cells of the murine macrophage cell line J774. AC were readily taken up. Once internalized, they remained in a resting state for several hours, whereas extracellular AC, e.g., in areas without macrophages, formed small networks of branched hyphae. Hence, phagocytosis clearly retarded AC germination.

After 6 h of infection, more than 70% of the AC resided in vacuoles that were stained with the LysoTracker DND26 dye, indicating an acidification and maturation of the uptake vesicles toward phagolysosomes. In minimal medium that was adjusted to pH 5, a value that was previously reported for J774 phagolysosomes (Kuehnel et al., 2001), AC showed a normal germination. This indicates that the observed delayed germination was not due to the acidified conditions, but may reflect that the phagosome is largely devoid of nutrients and that the spores have to cope with other antimicrobial effector mechanisms. After incubation of infected J774 macrophages for 24 h, many AC initiated germination, indicating that these spores were still viable and able to overcome the defense mechanisms of the phagocyte. Those AC that had formed germ tubes, exposed β-glucan antigens on their entire surface, most likely as a consequence of germination-associated cell wall rearrangements. Unexpectedly, we observed a release of galactomannan antigens from the surface of internalized AC. Such galactomannan-positive structures were detectable in the cytoplasm and below the cytoplasmatic membrane of infected macrophages. Hence, cell wall material is released from the AC surface and translocates from the phagolysosome to the cytoplasm. Whether these antigens are processed and able to trigger a response from the host cell remains to be determined. Published data implicated several receptors in the recognition of galactomannan (Garlanda et al., 2002; Serrano-Gómez et al., 2004; Chiodo et al., 2014; Steger et al., 2019), but more studies are clearly required to define the immunological relevance of the AC-associated galactomannan antigen.

In summary, AC from *A. terreus* can be well distinguished from PC by their strong and uniform galactomannan staining. The presence of galactomannan on the surface of AC agrees with a direct emergence from vegetative hyphae that also display galactomannan on their surface. AC and PC furthermore differ in their sensitivity to desiccation and in their speed of germination. The observed production of AC in infected tissues might contribute to the dissemination of *A. terreus* to secondary sites of infection, which is a severe complication especially in *A. terreus*-mediated invasive aspergillosis.

## Materials and Methods

### Strains and Culture Conditions

Strain At17-14 was isolated in the local pathology from the kidney of a dog. The isolate was grown on Sabouraud plates. DNA was isolated from a subculture of a single colony using the MasterPure Yeast DNA Purification Kit (Epicentre Biotechnologies, Madison, WI, USA). The ITS region was amplified using oligonucleotides ITS5 (5′-GAAGTAAAAGTCGTAACAAGG-3′) and Mas266 (5′-GCATTCCCAAACAACTCGACTC-3′) (Persinoti et al., 2018). In a second PCR reaction, we amplified a 1,132-bp fragment of the β-tubulin gene using oligonucleotides tub-For (5′-TGGTGCCGCTTTCTGGTA-3′) and tub-beta-REV2 (5′-AAGGAGTGGGCGCCAC-3′). Both PCR products were sequenced and homologous sequences in the database were identified using the Nucleotide BLAST algorithm at https://blast.ncbi.nlm.nih.gov/Blast.cgi.

SBUG844 is an environmental *A. terreus* isolate that was used in previous studies by Slesiona et al. (2012a,b). The *A. terreus* strain T9 was described by Jukic et al. (2017). Strain D141 is a clinical *A. fumigatus* isolate derived from an aspergilloma patient (Reichard et al., 1990). Cells of the murine J774 macrophage cell line were routinely grown in RPMI1640 medium supplemented with 5% fetal calf serum. For live-cell imaging, this medium was additionally buffered with 25 mM HEPES/pH 7.4.

### Isolation of AC

A volume of 40ml Sabouraud medium was placed in a 50-ml tube and inoculated with 7.5 × 10^5^ PC of the desired strain. Cultures were incubated at 37°C with gentle agitation (140 rpm). On each day, all parts of the mycelium that started to form a biofilm on top of the medium were removed to avoid PC formation and the residual mycelium was moved to the bottom of the tube using a sterile 10-ml pipette. On day 5, samples were vortexed to detach AC from hyphae and filtered through three layers of Miracloth (Merck, Darmstadt, Germany). After centrifugation (10,000 × *g*, 10min), the supernatant was carefully removed and the conidial pellet were resuspended in 10ml of sterile distilled water. After another washing step, the supernatant was removed, the pellet was resuspended in 1ml sterile, distilled water, and the spores were counted using a Neubauer chamber.

### Isolation of PC

The desired strains were cultivated on Sabouraud agar in cell culture bottles (T25 flasks, Sarstedt, Nümbrecht, Germany) and incubated for 3–4 days at 37°C. Conidia were harvested by shaking the bottle after the addition of 6–8ml sterile, distilled water + 0.01% Tween20, and 8–10 sterile glass beads per flask. The resulting suspension was filtered through a layer of Miracloth, and the concentration of conidia was determined using an Ultrospec10 cell density photometer (Amersham Biosciences, Amersham).

### Germination Assay

To determine the conidial diameters, freshly isolated AC and AC that were incubated in Sabouraud medium for 6 h at 37°C were analyzed using a DM750 microscope (Leica Microsystems, Wetzlar, Germany). Images were taken with an ICC50 W camera (Leica Microsystems), and conidial diameters were determined using the Leica Application Suite V4 software. The viability of the spores was checked microscopically after overnight incubation in Sabouraud medium at 37°C. To investigate the germination speed, 1ml of the indicated medium in a 24-well plate was inoculated with 4 × 10^5^ AC or PC and incubated at 37°C. At the desired time points, samples were fixed by the addition of 100 μl of 37% formaldehyde and analyzed using a DM IL LED microscope (Leica Microsystems). Images were taken with a Canon EOS600D camera. Using these images, cells were categorized as resting conidium, swollen conidium, germling, or hypha. Resting and swollen conidia were differentiated by their size, and germlings had a shorter (<20μm) and hyphae a longer (>20μm) filament. Representative images are shown in Supplementary Figure 4.

### Desiccation Assay

Freshly isolated AC and PC of At17-12 and PC of *A. fumigatus* strain D141 were suspended in distilled, sterile water and 4 μl were seeded in small droplets onto the surface of tissue culture Petri dishes (μ dish; IBIDI, Martinsried, Germany). The plates were desiccated for 16 h in 37°C. Sabouraud medium was added to the dry conidia and, as a control, to conidia from the original, non-desiccated spore suspension, and the plates were incubated for 14 h at 37°C. Images were taken using a Leica DM IL LED microscope equipped with a 10× objective and a Canon EOS 600D camera.

### Phagocytosis Assay

Freshly isolated AC were labeled overnight at 4°C on a rotary device by incubation in 0.1M carbonate buffer, pH 9.6 supplemented with 0.1 mg/ml FITC. Labeled conidia were washed three times in PBS, checked for homogenous green fluorescence, and counted. Nearly confluent layers of J774 cells grown on glass coverslips in a 24-well plate were infected with 1 spore per 2 cells. For inhibition experiments, cells were incubated with either laminarin (100μg/ml) or mannan (300μg/ml) (both Merck, Darmstadt, Germany) for 1 h. Subsequently, AC were added in medium containing the abovementioned concentrations of the respective inhibitor. The cultures were incubated at 37°C, and coverslips were fixed at the indicated time points using 3.7% formaldehyde/phosphate-buffered saline (PBS). Samples were stained with the mab L10-1 and analyzed using a Zeiss LSM 880 confocal laser scanning microscope (Carl Zeiss, Jena, Germany).

To evaluate whether AC and PC survive phagocytosis by J774-macrophages, confluent layers of J774 cells were grown on coverslips in a 24-well plate and infected with FITC-labeled conidia (one conidium per two macrophages) and incubated at 37°C. After 1 h, the medium was replaced with fresh medium containing 0.05μg/ml itraconazole to prevent fungal overgrowth. Samples were fixed after 6 and 24 h using 3.7% formaldehyde/PBS, stained with 25μM Calcofluor white/PBS for 5min, and analyzed by confocal scanning microscopy.

### Immunofluorescence Staining

Hyphae were grown on glass coverslips and fixed for 5min with 3.7% formaldehyde/PBS at the indicated time points. Samples were then washed with PBS and incubated with the primary monoclonal antibody for 30min at 37°C. The coverslips were washed three times with PBS and subsequently incubated with a Cy3-labeled anti-mouse IgG + IgM antibody. Samples were stained with 25μM Calcofluor white/PBS for 5 min at RT. Phalloidin-FITC was used to visualize the filamentous actin cytoskeleton of J774 cells. Samples were mounted with Vectashield Mounting Medium, and images were taken using a Zeiss LSM880 confocal laser scanning microscope equipped with a 63× objective.

### Detection of Acidified Phagosomes

For live-cell microscopy, J774 macrophage were grown in a 35-mm μ-dish (IBIDI, Gräfelfing, Germany) in HEPES-buffered cell culture medium (pH 7.4). LysoTracker DND-26 (Thermo Fisher) was added at a concentration of 1μM. After 30min at 37°C, cells were infected with one AC per two cells. For further incubation, the dish was transferred to a temperature-adjusted chamber (37°C) that was attached to a Zeiss LSM 880 microscope. Confocal images were taken at the indicated time points.

### TNFα Enzyme-Linked Immunosorbent Assay

Confluent monolayers of J774 macrophages in a 96-well plate were infected with AC or PC of At17-14 with 2 or 10 spores per macrophage, respectively, and incubated for 6 h at 37°C. Cell-free supernatants were harvested and the TNFα concentration was determined using the ELISA MAXTM Deluxe Set Mouse TNF-α (BioLegend, San Diego, USA). Analysis was carried out in triplicates using 25 μl of sample volume and a dilution of 1:10 according to the protocol of the manufacturer, followed by the addition of an avidin-HRP-labeled secondary antibody and TMB substrate solution. Absorbance reading was performed in a NanoQuant infinite M200 pro microplate reader (Tecan, Crailsheim, Germany) at 450 and 570 nm.

### Statistics

Samples were analyzed for significance using a two-tailed *t*-test supplied with the GraphPad Prism program.

## Data Availability Statement

The original contributions presented in the study are included in the article/Supplementary Material, further inquiries can be directed to the corresponding author/s.

## Author Contributions

IH, LS, MB, JL, and FE designed the experiments. IH, CK, LS, and FE performed the experiments. FE wrote the manuscript. IH, LS, MB, and JL critically read the manuscript. FE and JL organized the funding. All authors contributed to the article and approved the submitted version.

## Funding

This study was supported by a grant of the Wilhelm-Sander-Stiftung to JL and FE.

## Conflict of Interest

The authors declare that the research was conducted in the absence of any commercial or financial relationships that could be construed as a potential conflict of interest.

## Publisher's Note

All claims expressed in this article are solely those of the authors and do not necessarily represent those of their affiliated organizations, or those of the publisher, the editors and the reviewers. Any product that may be evaluated in this article, or claim that may be made by its manufacturer, is not guaranteed or endorsed by the publisher.

## Supplementary Material

Supplementary Figure 1 Staining of hyphae of the *A. terreus* strains T9 and SBUG844 with the galactomannan-specific antibody L10-1 (red) and Calcofluor white (blue). **(B,D)** are maximum intensity projections; **(A,C)** show the corresponding (single plane) brightfield images. The positions of selected AC are indicated by arrows. The bar in **(C)** represents 10μm and is valid for all panels.

Supplementary Figure 2 Comparison of the germination of two different types of conidia from *A. terreus* and PC of *A. fumigatus*. Germination of purified AC of strain At17-14 and PC of the *A. terreus* strains At17-14 and SBUG844 as well as the *A. fumigatus* strain D141 was compared in Sabouraud and cell culture medium [**(A,B)**, respectively]. After 1, 3.5, 7, and 9 h, samples were analyzed microscopically and the percentages of resting conidia, swollen conidia, germlings, and hyphae were determined. For each measurement, three groups with 100 conidia each were analyzed. Standard deviations are indicated.

Supplementary Figure 3 Staining of an AC of strain At17-14 with the galactomannan-specific mab L10-1 (red) and Calcofluor white (blue). **(A–G)** show a sequence of confocal optical planes. The corresponding maximum intensity project is depicted in **(H)**. Sites of emerging germ tubes are indicated by arrows in **(A,F)**. A germ tube that was initially formed is indicated by an arrow in **(D)**. The bar in H represents 5μm and is valid for all panels.

Supplementary Figure 4 Representative images depicting the different stages of the germination process that were differentiated in the quantitative analysis. The bar represents 10μm and is valid for all panels.
