# Supplementary figures and images for: Characterization of Aspergillus terreus Accessory Conidia and Their Interactions With Murine Macrophages
Source: Front Microbiol. 2022 Jun 16;13:896145. doi: 10.3389/fmicb.2022.896145 (PMC9245049; doi:10.3389/fmicb.2022.896145)

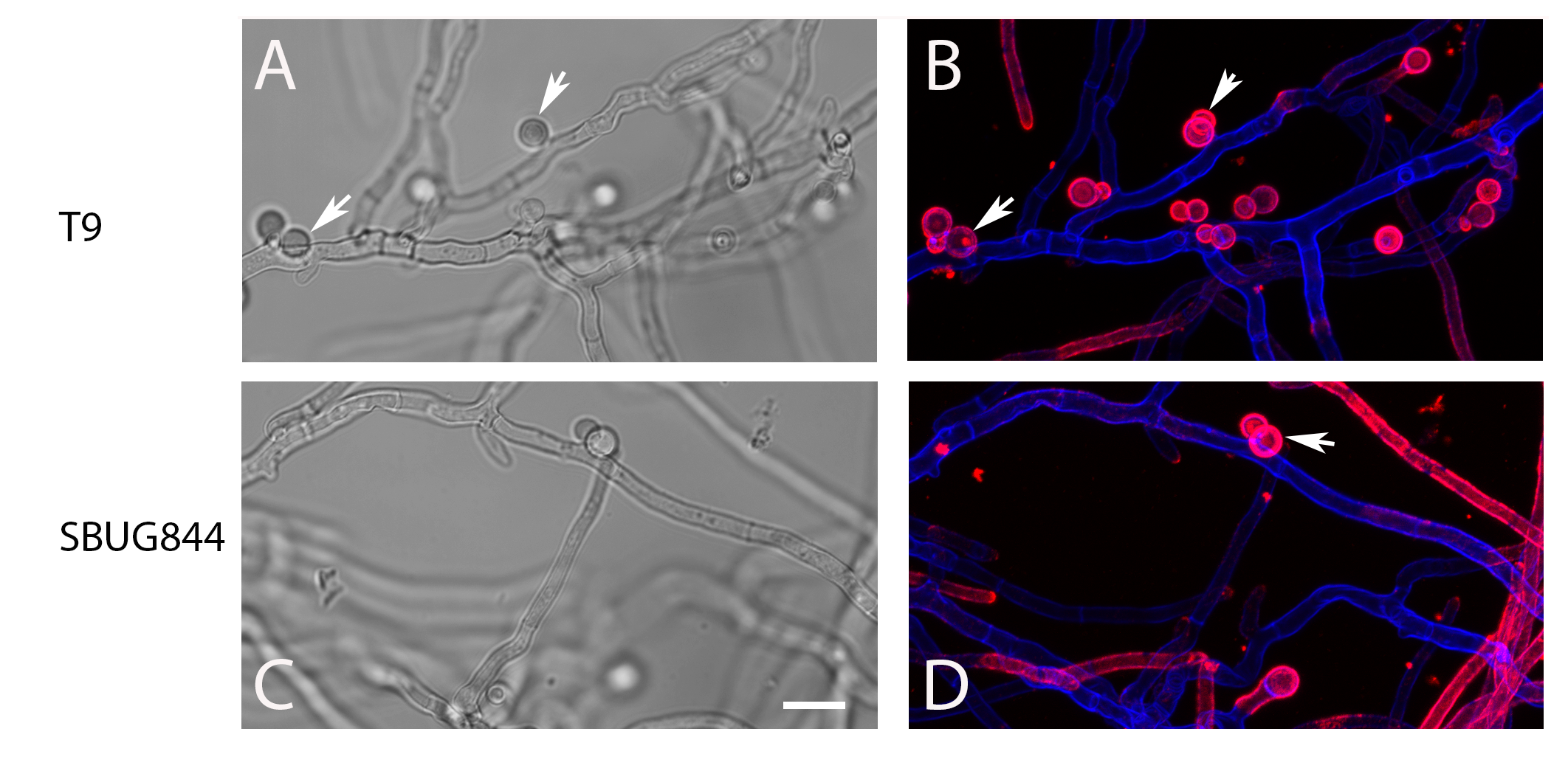

Supplement: Supplementary Figure 1 — Staining of hyphae of the A. terreus strains T9 and SBUG844 with the galactomannan-specific antibody L10-1 (red) and Calcofluor white (blue). (B,D) are maximum intensity projections; (A,C) show the corresponding (single plane) brightfield images. The positions of selected AC are indicated by arrows. The bar in (C) represents 10μm and is valid for all panels. [file Image_1.TIF]

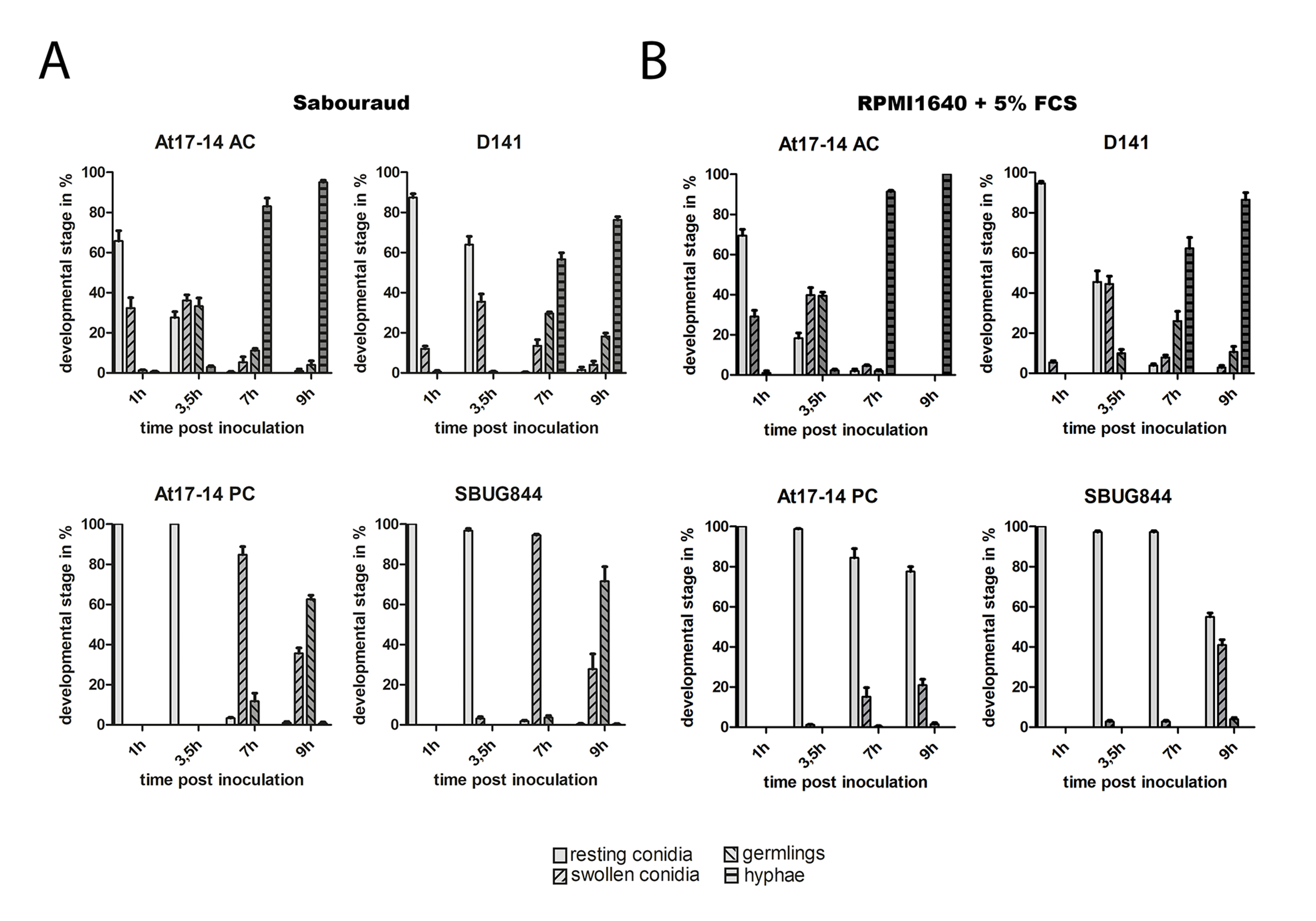

Supplement: Supplementary Figure 2 — Comparison of the germination of two different types of conidia from A. terreus and PC of A. fumigatus. Germination of purified AC of strain At17-14 and PC of the A. terreus strains At17-14 and SBUG844 as well as the A. fumigatus strain D141 was compared in Sabouraud and cell culture medium [(A,B), respectively]. After 1, 3.5, 7, and 9 h, samples were analyzed microscopically and the percentages of resting conidia, swollen conidia, germlings, and hyphae were determined. For each measurement, three groups with 100 conidia each were analyzed. Standard deviations are indicated. [file Image_2.TIF]

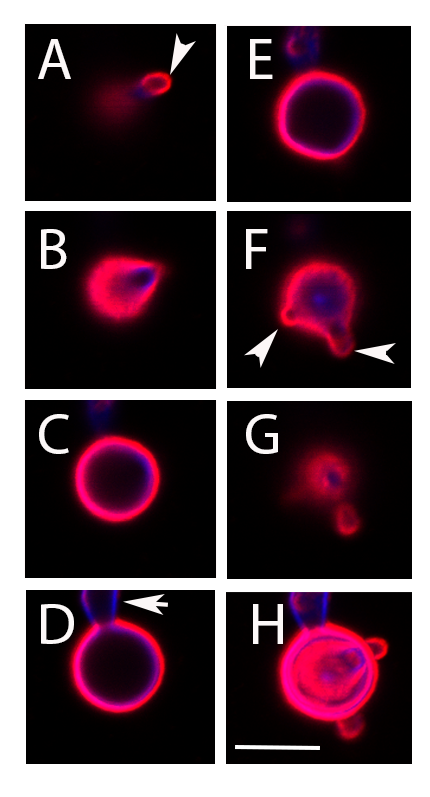

Supplement: Supplementary Figure 3 — Staining of an AC of strain At17-14 with the galactomannan-specific mab L10-1 (red) and Calcofluor white (blue). (A–G) show a sequence of confocal optical planes. The corresponding maximum intensity project is depicted in (H). Sites of emerging germ tubes are indicated by arrows in (A,F). A germ tube that was initially formed is indicated by an arrow in (D). The bar in H represents 5μm and is valid for all panels. [file Image_3.TIF]

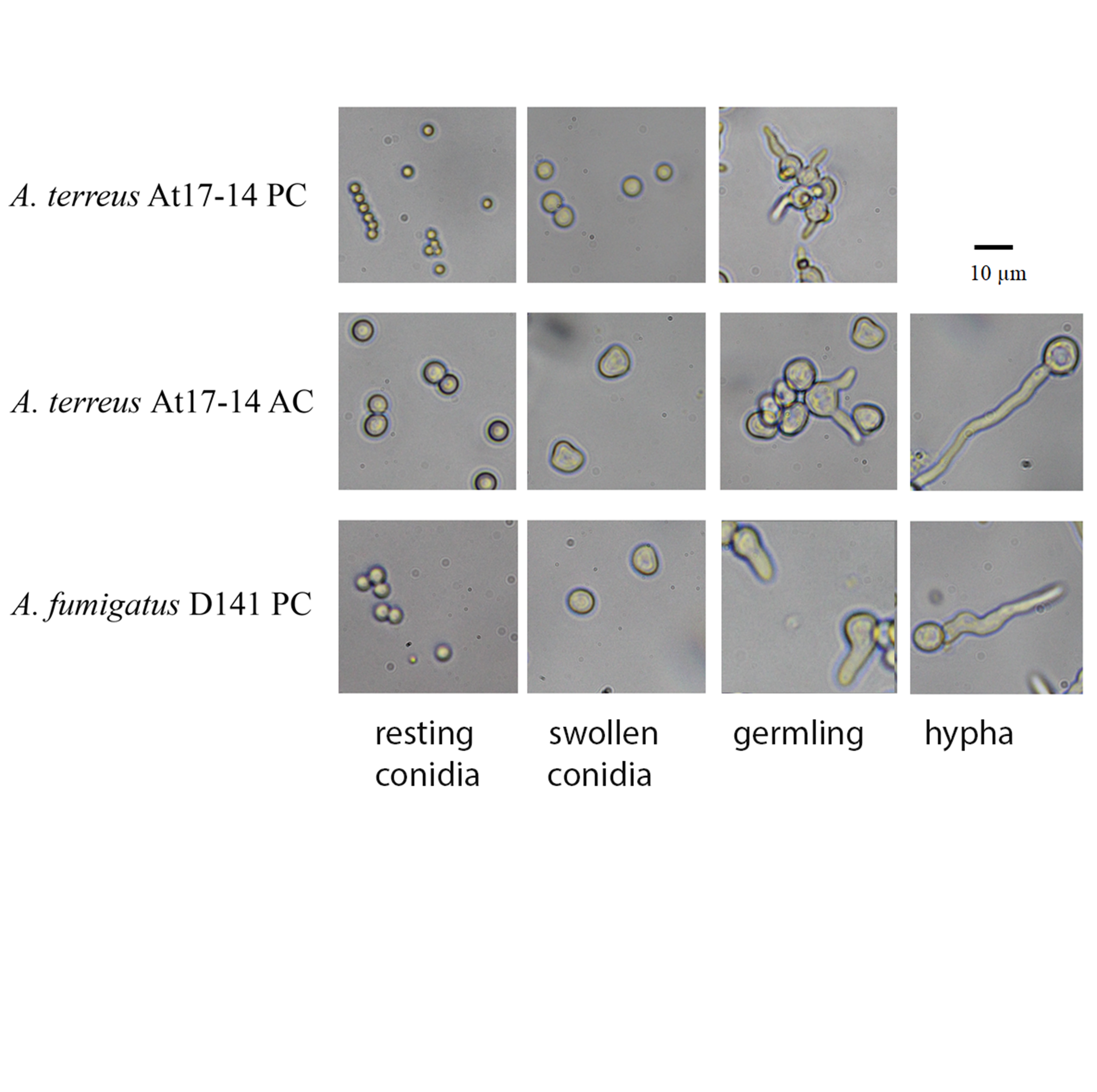

Supplement: Supplementary Figure 4 — Representative images depicting the different stages of the germination process that were differentiated in the quantitative analysis. The bar represents 10μm and is valid for all panels. [file Image_4.TIF]
